# Dynamic changes of natural killer cell immunophenotypes and receptors according to the mortality in the intra-abdominal murine sepsis model

**DOI:** 10.1186/s40635-025-00829-6

**Published:** 2025-11-19

**Authors:** Sang Hoon Han, Yeon-Mi Hong, Dayeong Kim, Eun Hwa Lee, Hye Seong

**Affiliations:** Division of Infectious Disease, Department of Internal Medicine, Yonsei University College of Medicine, 211 Eonju-ro, Gangnam-gu, Seoul, 06273 Republic of Korea

**Keywords:** Granzyme B, Immunophenotype, Ly49 family, Murine cecal ligation and puncture model, Natural killer cell, NK cell receptors, Sepsis

## Abstract

**Background:**

Sepsis is a life-threatening infectious syndrome that lacks targeted pharmacological therapies and poses major challenges in reducing mortality and long-term complications such as disability and frailty. Early and intensive intervention is critical to improving prognosis and preventing multiorgan dysfunction. However, alternative treatment strategies are urgently needed for patients who do not respond to guideline-based resuscitation, such as those outlined in the Surviving Sepsis Campaign. Natural killer (NK) cells are key effectors of the innate immune system, and their balanced activity may be crucial in preventing the progression of sepsis. Given conflicting evidence on whether NK cell activity (NKA) is protective or harmful, we investigated NKA in a murine model of intra-abdominal sepsis, assessing activating and inhibitory NK receptors (NKRs), as well as NK cell subsets in whole blood, bone marrow, lymph nodes, spleen, and liver.

**Methods:**

C57BL/6 mice underwent cecal ligation and puncture (CLP) to induce mid-grade (MGS, 30% 7-day survival) or high-grade sepsis (HGS, 0% 7-day survival), with sham-operated mice as controls. Blood and immune-related organs were collected on days 1, 3, and 7 post-surgery (MGS: days 1, 3, 7; HGS: days 1, 3; Sham: day 7). Flow cytometry was used to analyze CD11b and CD27 expression to define maturation-associated cytolytic and cytokine-producing NK cell phenotypes. CD3⁻NK1.1⁺ NK cells were purified by FACS for RT-PCR of activating (Ly49D, Ly49H) and inhibitory (Ly49C, Ly49G2) NKRs, and ELISA was performed for granzyme B and IFN-γ.

**Results:**

Our experiments consistently showed that in MGS, NKA—initially suppressed—was significantly restored by day 7 after CLP. This recovery was characterized by increased expression of activating NKRs, decreased inhibitory NKRs, expansion of terminally differentiated cytotoxic NK subsets (CD11b^+^/CD27^−^), higher total NK cell counts, and elevated granzyme B levels. In contrast, HGS, associated with high lethality, was marked by persistent suppression of NKA.

**Conclusions:**

The sustained impairment of NK cell phenotype is associated with lethal outcomes in sepsis.

**Supplementary Information:**

The online version contains supplementary material available at 10.1186/s40635-025-00829-6.

## Background

Sepsis syndrome, for which direct and specific drugs are currently unavailable, is a serious infectious condition leading to high mortality and long-term disabilities, irrespective of immediate intensive resuscitation, appropriate antimicrobial treatment, and immunomodulation with corticosteroids or immunoglobulins (Ig) [1–4]. The initial intricate pathophysiological alterations of sepsis-induced devastating multiorgan dysfunction are associated with exaggerated upregulation of the innate immune system, resulting in an overwhelming release of inflammatory mediators called cytokine storm [1, 3, 5–7]. Therefore, maintaining homeostasis of immune responses plays an important role in adequately controlling infection and inflammation and improving prognosis [6].

Natural killer (NK) cells are crucial cellular components of the rapid host defense mechanisms during early sepsis caused by various bacterial and viral pathogens [7–15]. Several human studies have shown that absolute NK cell counts or percentages of total lymphocytes in the early stage of sepsis are associated with gravity or short-term outcomes with conflicting (positive or negative) correlations [16–20]. In a mouse model of severe acute pneumonia caused by *Pseudomonas aeruginosa*, an apoptotic deficiency of spleen-resident NK cells resulted in earlier death without a change in the bacterial load in the lungs [21]. The gradually increasing number of liver- and spleen-resident NK cells after cecal ligation and puncture (CLP) produced higher serum anti-inflammatory interleukin (IL)-10 levels through the decreased IL-18 receptor, which ultimately caused mortality in a polymicrobial murine sepsis model [22]. In addition, NK cell-depleted mice had improved survival rates through lowering pro-inflammatory cytokines (e.g., interferon-γ [IFN-γ], IL-6) in the septic shock induced by *Streptococcus pyogenes* [14]. These data suggest that NK cells are significantly involved in the complex immunological mechanisms of incipient sepsis with close interactions with diverse inflammatory mediators; however, it is still not clear whether NK cell activity (NKA) could be comprehensively beneficial or harmful to the outcome of universal sepsis syndrome or sepsis in particular situations (e.g., basic host immune status and old age) or by specific microbes [9, 19, 23–27].

NKA is systematically regulated by an overall balance between signals delivered through many activating and inhibitory NK cell receptors (NKRs), which are classified into Ig and C-type lectin superfamilies [28, 29]. In a human study of intensive care unit participants, the killer cell Ig-like receptor-3DL1 (CD158e), an inhibitory NKR, was significantly higher in the sepsis group than in the systemic inflammatory response syndrome group; however, other activating or inhibitory NKRs, including NKp30, NKp46, and NKG2A/C/D, were similar between the two groups [20]. Little is known about NKA alterations based on the dynamic changes in NK cell subpopulations and NKRs in the blood and lymphoid organs during the initial stages of sepsis according to mortality. We conducted this study to identify the distinct features of NKA that cause early sepsis-attributable death by comparing time-specific differences in surface antigens and receptors among murine sepsis groups with different severity and mortality.

## Materials and methods

### Polymicrobial sepsis model in mice

**Fig. 1:**
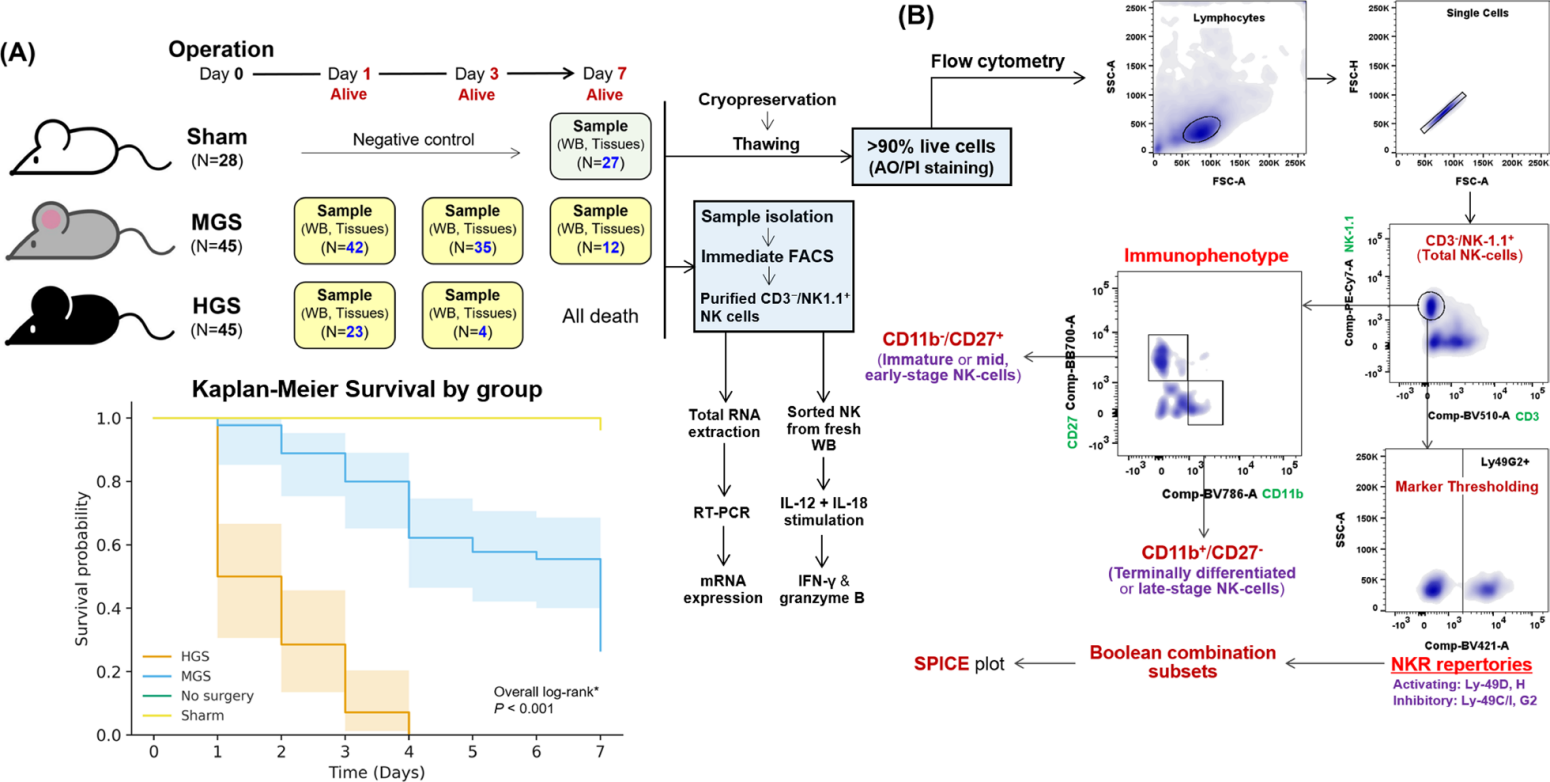
Overview of the experimental workflow using the CLP-induced sepsis mouse model, including subgroup allocation and the gating strategy applied for flow cytometric analysis. **A** Schematic overview of the experimental procedure illustrates the initial number of mice allocated to each group at the start of the experiment, along with the number of surviving mice on each designated postoperative day—corresponding to the number of samples collected. Notably, no samples were obtained on day 7 in the HGS group, as all mice in this group had died by that time. As negative controls, the sham group was used, in which mice underwent the same surgical procedures as the CLP group except for cecal ligation and puncture. In the MGS group, the cecum was ligated at a midpoint of the cecum corresponding to half the distance between the distal pole and the base of the cecum. In contrast, mice in the HGS group received ligation at approximately proximal 75% of the cecal length, indicating a more extensive ligation to induce higher grade sepsis. To confirm that the housing conditions were appropriate, three mice were observed for 7 days without undergoing any surgical intervention or experimental manipulation. The lower panel depicts the survival curves of each group over 7 days after surgery; the semi-transparent shaded bands around each step function denote the 95% CIs. In the Sham group, no deaths were observed during follow-up; therefore, the Greenwood variance is zero and the 95% CI collapses onto the survival curve, making the shaded band effectively invisible. The No-surgery group is not displayed because, after quality-control filtering, all records had zero events, leaving no analyzable observations and precluding Kaplan–Meier estimation. ^*^Pairwise log-rank tests (Holm-adjusted) indicated significant differences between all group pairs (*P* < 0.001), except Sham vs No-surgery, which was not significant. **B** Flow cytometry workflow presents the flow cytometry gating strategy, beginning with the identification of NK cell populations (CD3^−^/NK1.1^+^), followed by the classification of NK cell subpopulations (functional phenotypes according to maturation stage) based on the expression patterns of cell surface markers (CD11b and CD27). For each individual sample, flow cytometric analysis was performed with acquisition of a total of 1,000,000 cellular events. This panel also illustrates the subsequent analysis of the expression levels of four representative NKR within the gated NK cell population. The area between panels** A** and **B** illustrates a simplified workflow of sample processing from in vivo mouse models to subsequent downstream analyses. *AO* acridine orange, *CD* cluster of differentiation, *CI* confidence interval, *CLP* cecal ligation and puncture, *FACS* fluorescence-activated cell sorting, *HGS* high-grade sepsis, *IFN-γ* interferon-γ, *IL* interleukin, *Ly* lymphocyte antigen, *MGS* mid-grade sepsis, *mRNA* messenger RNA, *NK* natural killer cell, *NKR* NK cell receptor, *PI* propidium iodide, *RT-PCR* real-time polymerase chain reaction, *SPICE* simplified presentation of incredibly complex evaluations, *WB* whole blood

Murine experimental procedures were approved by the Institutional Animal Care and Use Committee (IACUC) of the Yonsei University College of Medicine (Approval No. 2020-0076). Intra-abdominal polymicrobial sepsis was induced using the CLP method as previously described [30]. Male C57BL/6 mice aged 8–10 weeks (Junbiotech Inc., Gyeonggi-do, South Korea) were randomly divided into three groups: (1) sham control (*N* = 28), (2) mid-grade sepsis (MGS) with 30–40% 7-day survival (*N* = 45), and (3) high-grade sepsis (HGS) with 100% mortality within 1 week after CLP (*N* = 45) (Fig. 1A). For MGS, the cecum was ligated at 50% of the distance from the distal pole to the cecal base (just distal to the ileocecal junction). For HGS, the proximal ~ 75% of the cecum was ligated. The ligated cecum was perforated at a single site by three through-and-through passes with a 21-gauge needle from the mesenteric toward the antimesenteric side, and the cecum was then gently compressed to extrude a small amount of fecal content [30]. The sham group underwent an identical laparotomy and cecal exteriorization without ligation or puncture. Sham cohorts were included as terminal controls and harvested on day 7 only, matched to septic endpoints (MGS/HGS), rather than as longitudinal baselines, in accordance with the prespecified design and the principles of the 3Rs (replacement, reduction, and refinement) [31]. All mice received a subcutaneous fluid bolus of pre-warmed normal saline for resuscitation and subcutaneous tramadol (Aju Pharm Co., Ltd., Seoul, South Korea) for analgesia (Fig. 1A).

Sampling time points (days 1, 3, and 7) were prespecified to capture early (hyperinflammatory), intermediate (transition), and late (immune dysfunction) phases of CLP sepsis, aligned with our survival-based endpoints and guided by established CLP protocols [32, 33]. Mice were euthanized by CO₂ inhalation as follows: three naïve mice maintained under normal conditions for 7 days; 27 mice at day 7 following sham surgery; 42, 35, and 12 mice at days 1, 3, and 7, respectively, in the MGS group; and 23 and 4 mice at days 1 and 3, respectively, in the HGS group. As all mice in the HGS group died within 7 days following CLP surgery, no samples were available from this group at day 7 (Fig. 1A).

In accordance with IACUC-approved protocols, all mice were monitored every 6–8 h for signs of septic deterioration, and humane endpoint criteria were strictly applied. If mice showed signs of imminent death—such as sustained hypothermia, unresponsiveness to stimuli, severe lethargy, or labored breathing—they were immediately euthanized by CO_2_ inhalation to prevent undue suffering. Therefore, death was not used as an experimental endpoint, and no animals were left to die spontaneously without intervention.

### Isolation of mononuclear cells from the whole blood, bone marrow, LNs, liver, and spleen

Whole blood (WB) was collected by cardiac puncture under anesthesia. Immediately afterward, mice were transcardially perfused with ice-cold phosphate-buffered saline (PBS) to eliminate residual circulating blood. The spleen, liver, lymph nodes (LNs), and bones (femur and tibia) were aseptically harvested for downstream analyses. Bone marrow (BM) cells were obtained by flushing the femur and tibia. Briefly, small pieces of bone epiphysis were placed in a microcentrifuge tube with a hole at the bottom, which was nested into a larger tube containing ice-cold PBS supplemented with 2% fetal bovine serum (FBS; Welgene Inc., Gyeongsangbuk-do, South Korea). The nested tubes were centrifuged for 3 min to flush out BM cells, and the resulting cell suspension was filtered through a 70-µm nylon mesh (Falcon^®^ Cell Strainers, Corning Inc., NY, USA) to remove debris [34].

The axillary/inguinal lymph nodes (LNs), spleen, and liver tissues were gently disrupted with a tissue grinder (KIMBLE^®^ KONTES^®^ Dounce, DWK Life Sciences, NJ, USA) and then further homogenized with the 200-µm nylon mesh sieve and pestles. Cell suspensions from the dissociated pieces were subsequently filtered through a 70-µm nylon mesh to obtain single-cell suspensions. The pelleted cells were resuspended in 40% isotonic Percoll^™^ PLUS (GE Healthcare Biosciences AB, Uppsala, Sweden), and the suspension was layered on 70% Percoll^™^ PLUS and then centrifuged for 30 min. Mononuclear cells in the interlayer were collected using a syringe and washed [22, 35]. Live cells isolated from WB, BM, LNs, spleen, and liver were resuspended in red blood cell (RBC) lysis buffer (BioLegend Inc., San Diego, CA, USA) and incubated on ice. After centrifugation, the pellet was repeatedly washed with ice-cold PBS until erythrocytes were visually depleted.

Cells obtained from all experimental samples were verified as mononuclear based on mean diameter (5–8 μm) measured with a cell viability analyzer (Cellometer^®^ Auto 2000, Nexcelom Bioscience, Lawrence, MA, USA). The mononuclear cells were cryopreserved at − 80 °C in 90% FBS with 10% dimethyl sulfoxide for flow cytometry. After thawing, cell viability was assessed using Acridine Orange/Propidium Iodide (AO/PI) staining with the LUNA^™^ Cell Viability Kit (Logos Biosystems, Gyeonggi-do, South Korea), followed by fluorescence microscopy (LUNA-FL^™^ Dual Fluorescence Cell Counter, Logos Biosystems). This method reliably distinguishes live (AO^+^, green) from dead (PI^+^, red) cells based on membrane integrity. Notably, the majority of isolated cells were lymphocytes and other immune cell populations, which are relatively resistant to cryopreservation-induced cell death. As a result, we consistently observed a high proportion of live cells—exceeding 90%—in our thawed single-cell preparations.

### Preparation of purified NK cells for downstream assays

To analyze messenger RNA (mRNA) expression of NK1.1, NKRs and granzyme B (GzmB), highly purified NK cells were isolated from WB and immunologically relevant tissues using fluorescence-activated cell sorting (FACS). Freshly isolated, non-cryopreserved mononuclear cells were prepared as single-cell suspensions following RBC lysis and washing with PBS supplemented with 2% FBS. Cells were stained with fluorochrome-conjugated monoclonal antibodies (mAbs) targeting CD3ε (BV510, 1:200 dilution) and NK1.1 (PE-Cy7, 1:200 dilution) in FACS buffer (PBS with 2% FBS and 2 mM EDTA) for 30 min at 4 °C in the dark. After staining, cells were washed twice and filtered through a 35 μm nylon mesh.

FACS was performed using a BD FACSAria^™^ III cell sorter (BD Biosciences, San Jose, CA, USA). The gating strategy included forward/side scatter profiling, doublet exclusion, and selection of live lymphocytes, followed by identification of CD3⁻/NK1.1⁺ NK cells. A total of 1000 viable NK cells were sorted per sample into RNase-free tubes containing lysis buffer and immediately processed for RNA extraction (Qiagen RNeasy^®^ Micro Kit, Qiagen, Hilden, Germany). This approach ensured a highly purified NK cell population, minimizing contamination from other immune cells and enabling accurate quantification of NK cell-specific gene expression.

### NK receptor panel and rationale

We profiled activating receptors Ly49D and Ly49H, together with inhibitory receptors Ly49C and Ly49G2, to interrogate complementary arms of NK regulation during sepsis. Ly49H is a canonical immunoreceptor tyrosine-based activation motif (ITAM)-coupled activating receptor that recognizes the murine cytomegalovirus (MCMV) glycoprotein m157 and signals through DNAX-activating protein 12 (DAP12) (and optimally DAP10) [36–38]. Beyond its established antiviral role, polymicrobial sepsis (CLP) induces quantitative loss and intrinsic signaling defects of Ly49H⁺ NK cells, including impaired DAP12 clustering and downstream activation, thereby providing a stringent readout of DAP12-dependent activation competence under septic inflammation [39, 40]. Ly49D serves as an additional DAP12-associated activator that engages stress- or MHC-I-related ligands, whereas Ly49C and Ly49G2 represent inhibitory, MHC-I-specific receptors integral to NK education/licensing and missing-self recognition [41, 42]. This receptor panel therefore captures both ITAM-mediated activation and immunoreceptor tyrosine-based inhibition motif (ITIM)-mediated inhibition, enabling systematic evaluation of the activation–inhibition balance across different severities of sepsis [40, 43, 44].

### Flow cytometry analyses

The cryopreserved cells were resuspended in PBS and incubated with a fluorochrome-conjugated mAbs cocktail (Table 1) in 100 µL of stain buffer (BD Biosciences) at 4 °C for 30 min in the dark. Flow cytometry experiments were performed on the BD LSRFortessa^™^ X-20 (BD Biosciences), and results were analyzed using FlowJo^™^ software (version 10.10, Ashland, OR, USA). The gating strategy was implemented as follows: (1) identification of the total NK population defined as NK1.1^+^ and CD3^−^ among lymphocytes, (2) compartmentalization of NK cell subsets in accordance with the fluorescent intensities of CD11b (integrin α-M or Mac-1) and CD27, corresponding to CD16 and CD56 in humans, respectively [45–49], and (3) measurement of NKR expression in total NK cells for Ly49D (Killer cell C-type lectin-like receptor [KLRA]-4) or Ly49H (KLRA-8) and Ly49C/I (KLRA-3/9) or Ly49G2 (KLRA-7) corresponding to activating and inhibitory NKR, respectively [50–54]. In order to evaluate the combinatorial expression patterns of these four NKRs, Boolean gating was performed using FlowJo^™^, based on the presence or absence of each receptor (Ly49C⁺/⁻, Ly49D⁺/⁻, Ly49G2⁺/⁻, and Ly49H⁺/⁻). The frequencies of each Boolean-defined subset were exported and visualized using SPICE (Simplified Presentation of Incredibly Complex Evaluations) software (v6.0, https://exon.niaid.nih.gov/spice/, NIH, Bethesda, MD, USA) (Fig. 1B) [55].

**Table 1:** Fluorochrome-conjugated monoclonal antibodies used for flow cytometry

Category	Fluorochrome	Clone	Cat. no	Supplies
Monoclonal antibodies				
Mouse anti-mouse NK1.1	PE-Cy7	PK136	552878	BD
Hamster anti-mouse CD3 epsilon	BV510	145-2C11	563024	BD
Rat anti-mouse CD11b	BV786	M1/70	740861	BD
Hamster anti-mouse CD27	BB700	LG.3A10	742135	BD
Mouse anti-mouse Ly49C and Ly49I	PE	5E6	553277	BD
Rat anti-mouse Ly49D	APC	eBio4E5 (4E5)	17-5782-82	Invitrogen
Rat anti-mouse Ly49G2	BV421	4D11	742879	BD
Mouse anti-mouse Ly49H	FITC	3D10	562536	BD
Isotype controls				
Mouse C3H x BALB/c IgG2a, κ	PE-Cy7	G155-178	557907	BD
Armenian hamster IgG1, κ	BV510	A19-3	563197	BD
Rat DA/HA IgG2b, κ	BV786	R35-38	563334	BD
Armenian hamster IgG1, κ	BB700	A19-3	566421	BD
Mouse 129/SvJ IgG2a, κ	PE	G155-178	553457	BD
Rat/IgG2a, κ	APC	eBR2a	17-4321-81	BD
Rat Fischer, CDF IgG2a, κ	BV421	R35-95	562602	BD
Mouse BALB/c IgG1, κ	FITC	X40	349041	BD

*APC* allophycocyanin, *BB* BD Horizon Brilliant Blue, *BV* BD Horizon Brilliant^™^ Violet, *Cat.* catalog, *CD* cluster of differentiation, *Cy* cyanine, *FITC* fluorescein isothiocyanate, *Ig* immunoglobulin, *Ly* lymphocyte antigen, *NK* natural killer cell, *No*. number, *PE* phycoerythrin

Both isotype-matched negative control and fluorescence minus one (FMO) control corresponding to each fluorochrome-conjugated mAb were used to exclude nonspecific binding and to establish objective gating thresholds for surface marker expression. This approach enabled accurate discrimination of true positive signals from background noise and improved the reliability of detecting low-expression markers.

### Quantitative real-time polymerase chain reaction (RT-PCR)

Total RNA was immediately extracted from the FACS-purified NK cells isolated from each sample using the guanidinium thiocyanate–phenol–chloroform extraction method (TRIzol^®^, Invitrogen, Thermo Fisher Scientific, Waltham, MA, USA). Reverse transcription was performed using a RevertAid^™^ First Strand cDNA Synthesis Kit (Thermo Fisher Scientific). The RT-PCR was implemented with a LightCycler^®^ 480 SYBR Green I Master qPCR mix kit (Roche Life Science, Penzberg, Germany) on the LightCycler^®^ 480 system (Roche Life Science) at 95 °C for 5 min followed by 45 cycles at 95 °C for 10 s and 60 °C for 30 s. Forward and reverse primers for NK1.1, Ly49C, Ly49D, Ly49G2, Ly49H, GzmB, and glyceraldehyde-3-phosphate dehydrogenase (GAPDH) were designed by Cosmo Genetech, Inc. (Seoul, South Korea) (Table 2). The quantitation of mRNA expression was determined using the comparative method 2^−ΔΔCt^, with normalization of the housekeeping gene *GAPDH*. The sham group served as the reference control for all fold-change calculations. RT-PCR was performed twice for each sample, and the average values were used in the final analysis.

**Table 2:** Primers used for real-time polymerase chain reaction

Genes	Sequence (5′ → 3′)	
	Forward	Reverse
NK1.1	GACACAGCAAGTATCTACCTCGG	TCAGAGCCAACCTGTGTGAACG
Ly49C	GAGAACAGGACAGATGGGACAG	GCAGTTCGCTTTACATCCACTCC
Ly49D	AGATGAGGCTCAAGGAGACACG	TGTCATAAGCACTGCAACAGTTAC
Ly49H	GAATCCTCTGTTCCCTTCGGCT	GCAGTTATGGCGGTGGTTGAGA
Ly49G2	CACAAAAGACCCATCTCCAAGGC	GCAGGTCTGTTTACATCCACTCC
Granzyme B	ACTTTCGATCAAGGATCAGCA	GGCCCCCAAAGTGACATTTAT
GAPDH	CATCACTGCCACCCAGAAGACTG	ATGCCAGTGAGCTTCCCGTTCAG

*GAPDH* glyceraldehyde 3-phosphate dehydrogenase, *Ly* lymphocyte antigen, *NK* natural killer cell

### Granzyme B and IFN-γ quantification in stimulated cell supernatants

FACS-purified CD3^−^/NK1.1^+^ NK cells from fresh WB were cultured in RPMI 1640 medium (Welgene Inc.,) supplemented with 10% FBS and stimulated with recombinant mouse IL-12 (p70) (10 ng/mL; Invitrogen) and IL-18 (50 ng/mL; Invitrogen) for 16 h; to align the measurement with our biological question, we prioritized a secretion-based readout and therefore analyzed culture supernatants for GzmB and IFN-γ by enzyme-linked immunosorbent assay (ELISA). ELISA kits were used as follows: GzmB (Invitrogen) and IFN-γ (LEGEND MAX^™^, BioLegend, Inc.). The standard range of the GzmB and IFN-γ kits is 0.7–480 pg/mL and 15.6–1000 pg/mL, respectively. Absorbance was measured at 450 nm on a Multiskan SkyHigh microplate spectrophotometer (Thermo Fisher Scientific). All samples were assayed in duplicate and quantified using a standard curve.

### Statistical analysis

All data are presented as median and interquartile range (IQR). All graphs representing quantitatively measured data are depicted as box-and-whisker plots using the Tukey method. The central line within the box represents the median (Quartile 2), while the lower and upper edges of the box correspond to the 25th (Q1) and 75th (Q3) percentiles, respectively, with the IQR defined as Q1–Q3. The whiskers extend to values within the range of Q1 − 1.5 × IQR and Q3 + 1.5 × IQR, where IQR is the difference between Q3 and Q1. Data points outside this range are displayed as individual dots, indicating statistical outliers. One-way analysis of variance (ANOVA) was used for comparisons among the three groups. When the omnibus test was significant, pairwise between-group comparisons were conducted with Bonferroni correction. Comparisons between two independent groups were conducted using the two-sample *T*-test. A two-tailed *P*-value of ≤ 0.05 was considered statistically significant. A *P*-value < 0.01 was denoted by one asterisk, and a *P*-value < 0.001 by two asterisks. All statistical analyses were conducted using SPSS software (version 16, IBM^®^ SPSS^®^ Statistics Inc., Chicago, IL, USA), and visualization was generated using Prism (version 8.4.3, GraphPad^®^ Software Inc., La Jolla, CA, USA).

## Results

### Changes in NK1.1 expression and total NK cell counts

We established MGS and HGS sepsis models in C57BL/6 mice and collected WB, LNs, BM, spleen, and liver samples on days 1, 3, and 7 post-surgery to investigate temporal and severity-dependent changes in NK cell responses. Sham-operated mice served as controls (*N* = 27 on day 7). In the MGS group, samples were available at all time points (D1: 42, D3: 35, D7: 12), while in the HGS group, limited survival restricted sample collection to days 1 (*N* = 23) and 3 (*N* = 4). Flow cytometry was used to assess total NK cells (CD3⁻/NK1.1⁺), maturation markers (CD11b, CD27), and activating (Ly49D, Ly49H) and inhibitory (Ly49C, Ly49G2) NKRs. Expression of these NKRs at the mRNA level was quantified from FACS-purified NK cells (Fig. 1A; see Table S1 for details).

**Fig. 2:**
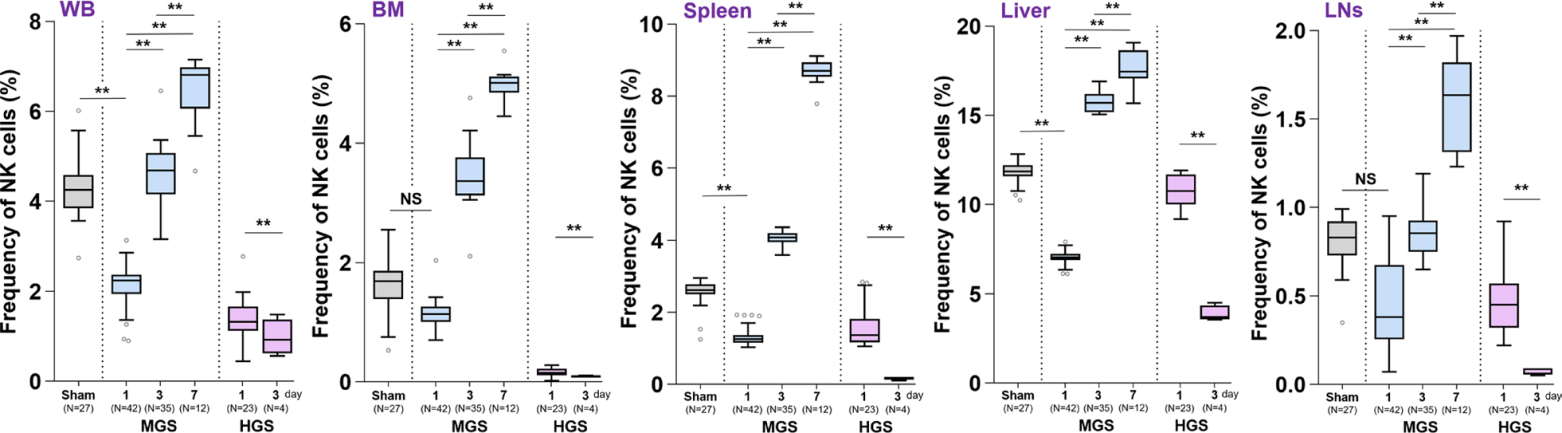
Dynamic alterations in NK cell populations over time in relation to sepsis severity. A quantitative comparison is shown for NK cell (CD3^−^/NK1.1^+^) frequencies among mononuclear cells (lymphocytes) across different time points in MGS and HGS groups. For objective quantitative analysis and valid statistical comparison, a total of 1 × 10⁶ events were acquired per sample in each flow cytometric experiment. After sequential gating, 100,000 lymphocytes were uniformly selected, and NK cell frequency was calculated as the proportion of CD3⁻/NK1.1⁺ cells within this lymphocyte population. Sample numbers for each group are shown in the figure. The sham group yielded 27 samples. In the MGS group, 42, 35, and 12 samples were collected on days 1, 3, and 7 post-CLP, respectively. In the HGS group, 23 and 4 samples were obtained on days 1 and 3; no samples were available on day 7 due to complete mortality. Data are displayed as box-and-whisker plots; dots indicate statistical outliers. Details of statistical procedures and box-plot conventions are provided in the Methods. Statistical comparisons were performed using two-way ANOVA with Bonferroni post hoc correction in the MGS group and two-sample T-test in the HGS group. ^*^P < 0.01, ^**^P < 0.001, NS, not significant. *ANOVA* Analysis of Variance; *BM* bone marrow, *CD* cluster of differentiation, *CLP* cecal ligation and puncture, *HGS* high-grade sepsis, *IQR* interquartile range, *LNs* lymph nodes, *MGS* mid-grad sepsis, *mRNA* messenger RNA, *NK* natural killer cell, *WB* whole blood

NK1.1 mRNA and NK cell frequencies were acutely suppressed on day 1 in both sepsis groups compared to sham, with more pronounced and persistent suppression in the HGS group. In MGS, NK1.1 mRNA levels gradually rebounded over time, surpassing sham levels by day 7 across all tissues (all *P* < 0.001), indicating recovery of NK cell populations. In contrast, HGS mice showed continued reduction in NK1.1 expression on day 3, consistent with a severity-driven impairment in NK cell restoration (Fig. 2).

Notably, NK1.1 expression in MGS-derived BM and LNs remained stable on day 1 but significantly increased on days 3 and 7 (all *P* < 0.001), paralleling the recovery seen in WB, spleen, and liver (all *P* < 0.001). Flow cytometry confirmed these trends, with NK cell frequencies decreasing on day 1 in both groups, followed by progressive restoration in MGS but further decline in HGS on day 3. The most consistent and prominent changes were observed in WB and liver samples (Fig. 2, Fig. S1).

### Differences of CD11b- or CD27-expressing NK cell subpopulations according to sepsis lethality

To determine whether sepsis severity influences the distribution of NK cell subpopulations—defined by CD11b and CD27 expression, which reflect maturation status and functional diversity—we analyzed their proportions in both MGS and HGS models. In the MGS group, the percentage of CD11b⁺/CD27⁻ terminally differentiated, cytolytic NK cells progressively increased on days 3 and 7 across all tissues examined, including WB, BM, LNs, spleen, and liver (all *P* < 0.001), suggesting active maturation toward cytolytic phenotypes in the context of moderate sepsis. In contrast, this subset was significantly reduced on day 3 in the HGS group, reflecting a failure to acquire terminal NK cell features under lethal inflammatory conditions.

Conversely, immature, cytokine-producing CD11b⁻/CD27⁺ NK cells progressively declined in the MGS group (all *P* < 0.001), whereas their sustained presence or increase in the HGS group indicates that HGS may hinder NK cell maturation and promote the accumulation of immature subsets (Table 3 and Fig. S3).

**Table 3:** Quantitative analysis of the proportions of CD11b or CD27 single-positive NK cell subsets in MGS and HGS groups

Specimen		MGS				HGS		
		1 d	3 d	7 d	*P*-value^a^	1 d	3 d	*P*-value^b^
WB	CD11b^+^/CD27^−^	19.6 (17.3–22.5)	57.3 (52.1–62.3)	86.1 (75.2–91.8)	< 0.001	31.0 (23.9–37.7)	11.9 (8.2–14.6)	< 0.001
	CD11b^−^/CD27^+^	36.2 (27.8–40.6)	19.6 (15.6–22.9)	7.58 (5.47–9.31)	< 0.001	17.8 (14.5–20.3)	34.7 (29.8–39.2)	< 0.001
BM	CD11b^+^/CD27^−^	59.3 (55.7–63.8)	70.8 (65.2–74.8)	91.5 (84.6–94/1)	< 0.001	63.7 (59.5–68.1)	25.2 (19.9–28.7)	< 0.001
	CD11b^−^/CD27^+^	33.0 (30.6–37.1)	14.8 (12.1–15.7)	7.21 (4.97–9.87)	< 0.001	29.1 (23.8–34.5)	53.1 (49.7–57.0)	< 0.001
Spleen	CD11b^+^/CD27^−^	15.1 (11.2–19.3)	25.2 (21.9–28.9)	60.3 (56.7–65.2)	< 0.001	37.8 (33.1–43.7)	16.0 (13.7–20.0)	< 0.001
	CD11b^−^/CD27^+^	39.7 (35.5–44.2)	18.7 (14.8–23.6)	13.1 (10.7–16.5)	< 0.001	23.0 (19.8–27.1)	59.3 (52.7–63.9)	< 0.001
Liver	CD11b^+^/CD27^−^	26.0 (22.1–31.7)	33.2 (30.8–36.5)	41.2 (37.9–45.7)	< 0.001	52.7 (49.0–56.5)	19.6 (17.1–22.0)	< 0.001
	CD11b^−^/CD27^+^	50.9 (45.8–56.0)	41.3 (38.8–43.6)	30.5 (27.7–34.1)	< 0.001	18.6 (15.2–20.9)	50.5 (45.2–56.3)	< 0.001
LNs	CD11b^+^/CD27^−^	2.10 (1.75–3.92)	15.3 (12.8–18.7)	44.9 (40.7–50.0)	< 0.001	29.7 (26.2–32.8)	6.7 (4.3–9.1)	< 0.001
	CD11b^−^/CD27^+^	75.8 (72.1–82.6)	6.8 (2.7–12.6)	2.7 (1.9–3.8)	< 0.001	19.5 (15.3–23.1)	40.7 (36.9–43.1)	< 0.001

All data obtained by flow cytometry are expressed as median (IQR). For each sample, 1,000,000 total events were acquired per analysis, and the percentages were calculated based on 5,000 gated CD3⁻/NK1.1⁺ lymphocytes. ^a^Comparisons among the day 1, 3, and 7 subgroups within the MGS group were performed using one-way ANOVA, followed by Bonferroni correction for post hoc pairwise comparisons. All statistical comparisons adjusted by Bonferroni correction yielded *P*-values < 0.001. ^b^Comparisons between day 1 and day 3 in the HGS group were conducted using the two-sample T-test. *ANOVA* analysis of variance, *BM* bone marrow, *CD* cluster of differentiation, *HGS* high-grade sepsis, *IQR* interquartile range, *LNs* lymph nodes, *Ly* lymphocyte antigen, *MGS* mid-grad sepsis, *NK* natural killer cell, *WB* whole blood

These findings demonstrate distinct NK cell maturation trajectories depending on sepsis severity: progressive maturation and cytotoxic competency in MGS, versus maturation arrest and accumulation of immature NK cells in HGS.

### Dynamic alteration of NK receptors in the MGS and HGS

We hypothesized that sepsis severity would differentially affect temporal expression patterns of activating and inhibitory NKRs, and analyzed their transcriptional dynamics in MGS and HGS models. In the MGS group, inhibitory receptors Ly49C and Ly49G2 mRNA levels were transiently elevated on day 1 in WB-, LNs-, and BM-resident NK cells compared to the sham group (all *P* < 0.001), but declined significantly on days 3 and 7 (*P* < 0.001 vs. day 1). In contrast, the HGS group showed a persistent and further increase in these inhibitory NKR transcripts on day 3 (all *P* < 0.001), suggesting sustained elevation under severe septic condition.

**Fig. 3:**
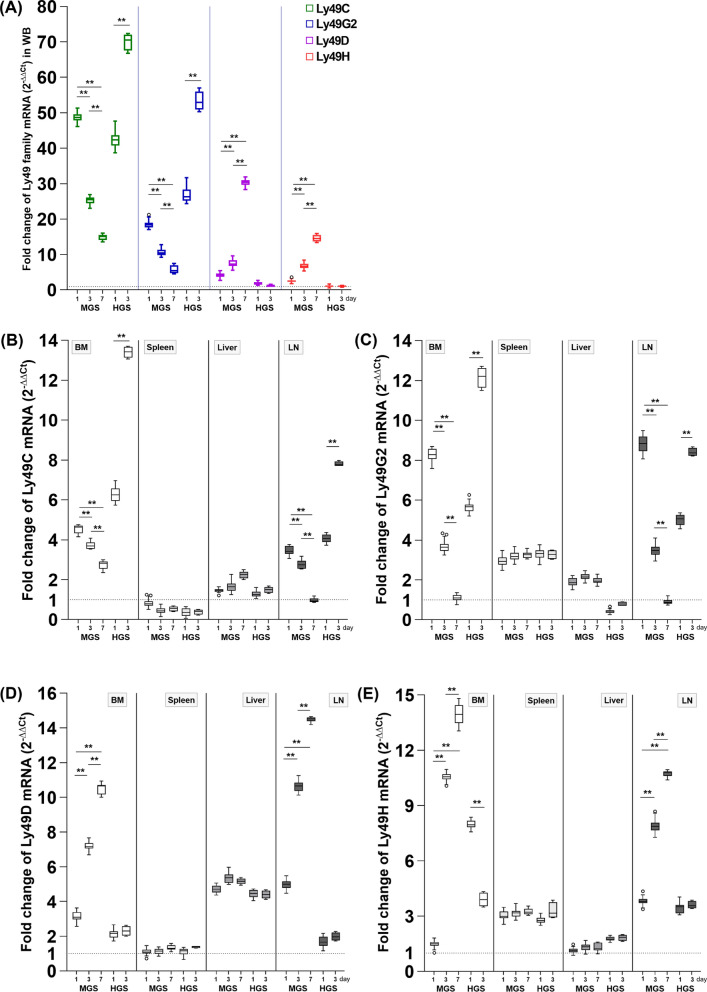
Differential mRNA expression of inhibitory and activating NK cell receptors in a murine model of mid-grade and high-grade sepsis. Relative mRNA expression levels were calculated using the 2^−ΔΔCt^ method, with the sham group (*N* = 27) serving as the reference control. In each panel, the dotted line on the Y-axis indicates a 2^−ΔΔCt^ value of 1, representing the baseline mRNA expression level in the sham group, which serves as the standard for fold-change comparisons. **A** Comparison of the expression levels of four Ly49 family (Ly49C, G2, D, and H) in the same WB samples. **B**, **C** Expression levels of inhibitory NKR (Ly49C and Ly49G2) in BM cells, LNs, spleen, and liver tissues. **D**, **E** Expression levels of activating NKR (Ly49D and Ly49H) in BM cells, LNs, spleen, and liver tissues. All RT-PCR experiments were performed using total RNA extracted from an equal number of purified 2000 NK cells isolated from each sample by FACS. The mRNA samples with an amount greater than 20 ng were used for subsequent RT-PCR analysis of NKR expression. For each experiment, two RT-PCR reactions were performed using the same total RNA extracted from a single sample, and the median value was used for analysis. Each graph is shown as a box-and-whisker plot using the Tukey method. Data points represented as circles indicate statistical outliers. In the MGS group, 42, 35, and 12 samples were collected on days 1, 3, and 7 after CLP surgery, respectively. In the HGS group, 23 and 4 samples were collected on days 1 and 3, respectively; no samples were available on day 7, as all mice in this group had died by that time. Statistical comparisons were performed using two-way ANOVA with Bonferroni post hoc correction in the MGS group and two-sample T-test in the HGS group. ^*^P < 0.01, ^**^P < 0.001. *ANOVA* analysis of variance, *BM* bone marrow, *CLP* cecal ligation and puncture, *Ct* cycle threshold value, *FACS* fluorescence-activated cell sorting, *HGS* high-grade sepsis, *IQR* interquartile range, *LNs* lymph nodes, *Ly* lymphocyte antigen, *MGS* mid-grade sepsis, *mRNA* messenger RNA, *NK* natural killer cell, *NKR* NK cell receptor, *RT-PCR* real-time polymerase chain reaction, *W*B whole blood

Activating receptors Ly49D and Ly49H were markedly increased in WB-, BM-, and LNs-resident NK cells on days 3 and 7 after MGS (all *P* < 0.001), whereas no significant change occurred in HGS between days 1 and 3. Notably, the expression of all four receptors remained unchanged in the spleen and liver across both groups and all time points (Fig. 3).

**Fig. 4:**
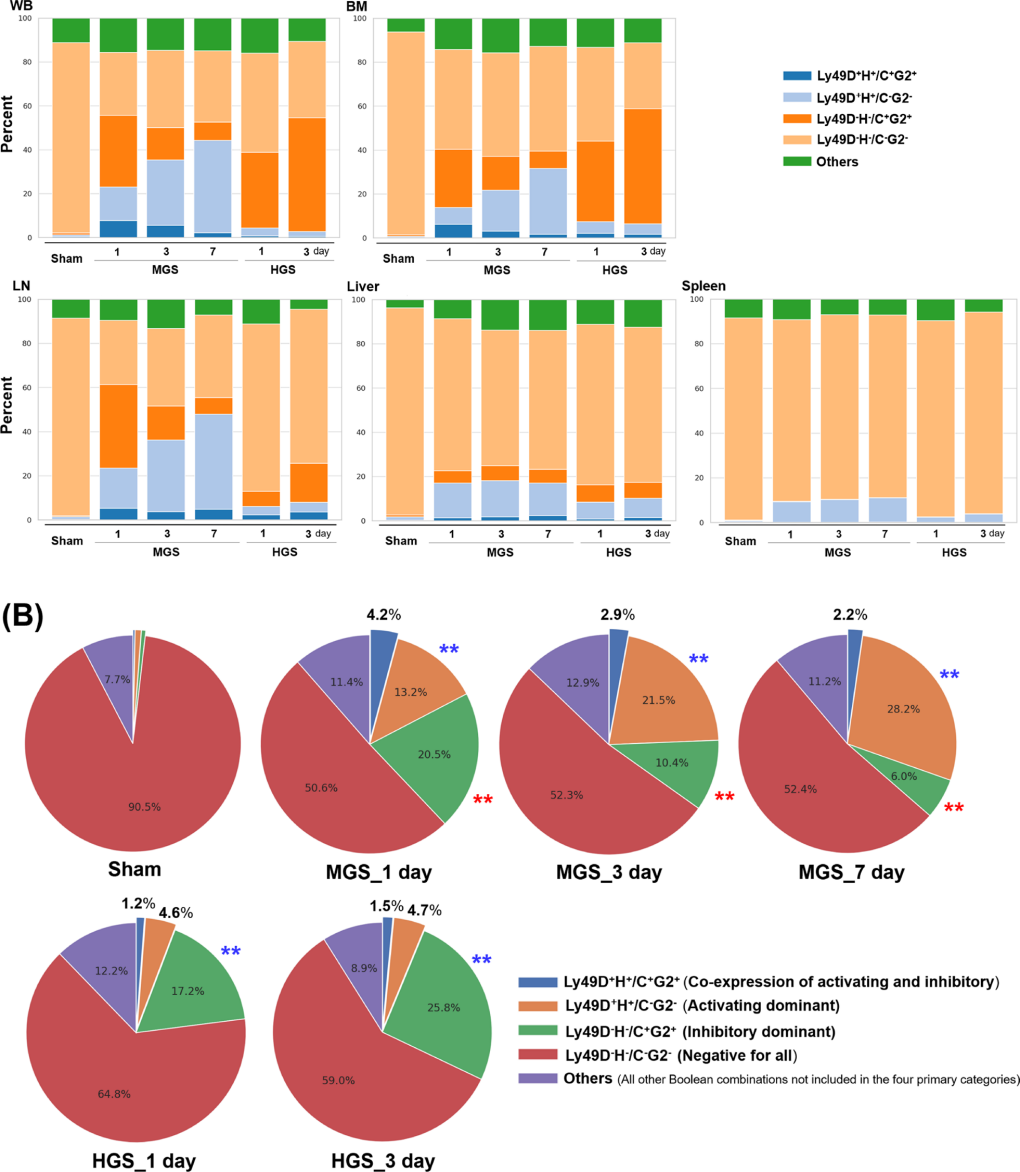
Boolean combination profiles of receptor-positive NK cell subsets across groups stratified by sepsis severity and time after sepsis induction. Boolean combinations of NK cell receptor expression were classified into five categories: (1) Ly49D^+^Ly49H^+^Ly49C^+^Ly49G2^+^ (co-expression of activating and inhibitory), (2) Ly49D^+^Ly49H^+^Ly49C^−^Ly49G2^−^ (activating-dominant), (3) Ly49D^−^Ly49H^−^Ly49C^+^Ly49G2^+^ (inhibitory-dominant), (4) Ly49D^−^Ly49H^−^Ly49C^−^Ly49G2^−^ (negative for all), and (5) Others (all other Boolean combinations not included in the four primary categories). **A** Stacked bar plots show the proportions of NK cell subsets defined by these Boolean categories across six groups stratified by sepsis severity (Sham, MGS, HGS) and time points (day 1, 3, or 7). Each bar represents the median percentage of NK cells expressing a given Boolean receptor combination in each group. **B** Pie charts illustrate the average distribution of Boolean-defined NK cell subsets across all individual samples within each group. For Boolean analysis, the frequency of each receptor combination was first calculated for each sample, and the group-level average was visualized. Red asterisks indicate a statistically significant decrease compared to the Sham group (*P* < 0.001), while blue asterisks indicate a significant increase within the same sepsis severity group (i.e., among MGS or among HGS) (*P* < 0.001). Data are presented as SPICE-style compositional plots, allowing a comparative overview of phenotypic shifts in NKR expression in response to varying degrees of intra-abdominal polymicrobial sepsis. *BM* bone marrow, *HGS* high-grade sepsis, *LNs* lymph nodes, *Ly* lymphocyte antigen, *MGS* mid-grad sepsis, *NK* natural killer cell, *NKR* NK cell receptor, *SPICE* simplified presentation of incredibly complex evaluations, *WB* whole blood

These transcriptional patterns were mirrored by Boolean flow cytometric analysis, which revealed parallel shifts in NK cell subsets defined by NKR combinations (Fig. 4A, B). Conventional gating also confirmed similar trends in individual NKR-positive populations (Fig. S4).

### Expression of granzyme B and IFN-γ

**Fig. 5:**
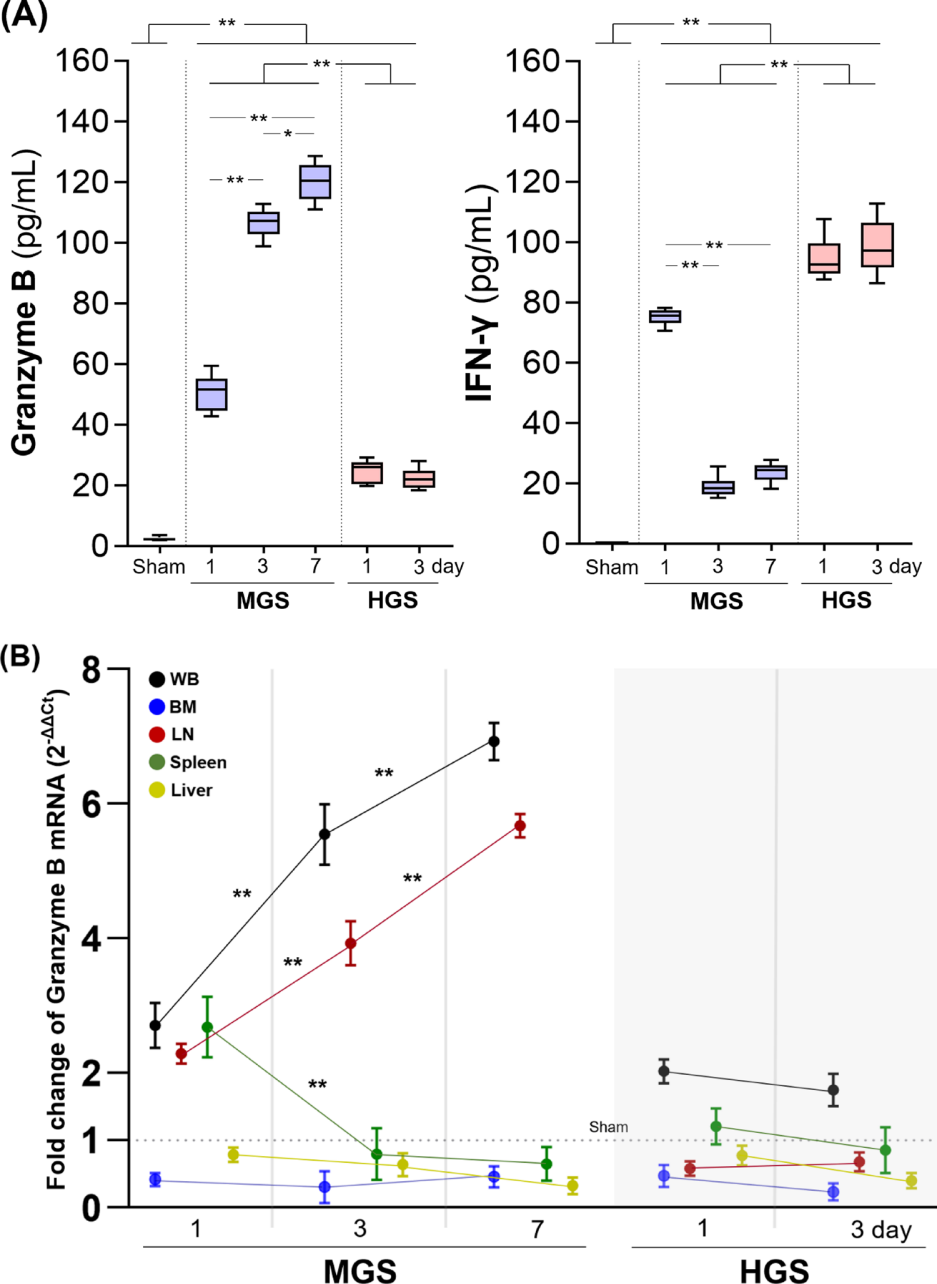
Plasma concentrations of granzyme B and interferon-γ, and relative mRNA expression of granzyme B in various organs. **A** Granzyme B and interferon-γ levels were measured in supernatants of NK cells cultured with IL-12 and IL-18 stimulation. For all groups (Sham, MGS, and HGS), each individual sample was measured in duplicate, and the median of the two values was used as the representative value for that sample. Each graph is shown as a box-and-whisker plot using the Tukey method. The Sham group consisted of 27 samples. In the MGS group, 42, 35, and 12 samples were collected on days 1, 3, and 7 after CLP surgery, respectively. In the HGS group, 23 and 4 samples were collected on days 1 and 3, respectively; no samples were available on day 7, as all mice in this group had died by that time. **B** Relative mRNA expression of granzyme B. In the sham group, 27 samples were measured in duplicate, and all values were below the detection limit. The mRNA expression levels obtained by real-time RT-PCR were normalized using the 2^−ΔΔCt^ method, with sham group values serving as the reference (baseline = 1.0). The dotted line on the Y-axis indicates a 2^−ΔΔCt^ value of 1, representing the baseline mRNA expression in the sham group for fold-change comparison. Statistical analyses were performed using two-way ANOVA followed by Bonferroni post hoc correction in the MGS group and two-sample T-test in the HGS group. ^*^P < 0.01, ^**^P < 0.001. *ANOVA* analysis of variance, *BM* bone marrow, *CLP* cecal ligation and puncture, *Ct* cycle threshold value, *HGS* high-grade sepsis, *IL* interleukin, *LNs* lymph nodes, *MGS* mid-grade sepsis, *mRNA* messenger RNA, *NK* natural killer cell, *RT-PCR* real-time polymerase chain reaction, *WB* whole blood

Given that GzmB and IFN-γ are key mediators of NK cell cytotoxicity and immunoregulation, we evaluated their functional output in MGS and HGS models. Upon IL-12/IL-18 stimulation, NK cells from both MGS and HGS mice secreted significantly higher levels of GzmB and IFN-γ compared to sham (all *P* < 0.001). In MGS, GzmB levels increased progressively from day 1 to 7, while IFN-γ levels declined after day 1. In contrast, HGS mice showed persistently low GzmB and elevated IFN-γ levels on days 1 and 3, with no significant changes between time points. Notably, GzmB levels in HGS were consistently lower, and IFN-γ levels consistently higher, than those in MGS at all comparable time points (all *P* < 0.001) (Fig. 5A).

Consistent with these findings, GzmB mRNA expression in the MGS group increased over time in WB and LNs (two- to sevenfold), decreased in the spleen after an initial peak, and remained unchanged in BM and liver. In the HGS group, GzmB mRNA remained consistently below sham levels across all tissues, with only a modest increase in WB (Fig. 5B).

## Discussion

Our study systematically delineates dynamic alterations in NKA across WB and lymphoid tissues following sepsis of varying severity. The key findings are as follows: (1) NK cell proportions among mononuclear cells declined acutely after sepsis onset regardless of severity, but only recovered over time in MGS, not in the fatal HGS model; (2) terminally differentiated, cytolytic NK cells (CD11b⁺/CD27⁻) expanded steadily after MGS [47–49, 56], whereas immature, cytokine-producing NK cells (CD11b⁻/CD27⁺) showed reciprocal trends—declining after MGS but increasing after HGS [56], suggesting disrupted maturation under lethal conditions; (3) activating NKRs (Ly49D/H) were gradually induced in MGS but remained blunted in HGS, while inhibitory NKRs (Ly49C/G2) declined persistently after a transient surge in MGS and were persistently elevated in HGS; and (4) consistent with these maturation and NKR changes, GzmB production increased progressively in MGS but remained low in HGS, suggesting impaired cytolytic potential under lethal septic conditions.

These immunophenotypic alterations prompted us to examine whether the observed changes in NKA could reflect prognostic differences and clinical significance in sepsis outcomes. Together with these coherent data from diverse examinations, early alterations in GzmB production and cytotoxicity against infected cells reflect dynamic shifts in overall NKA that vary with sepsis lethality, as follows [13, 27, 57]: (1) NKA is transiently suppressed after sepsis with lower predicted short-term mortality but gradually recovers; (2) in fatal cases, NKA remains persistently impaired; and (3) restored NKA in milder cases does not provoke hypercytokinemia. This inverse relationship between NKA and early sepsis mortality suggests its potential as a prognostic biomarker. Measurement of NKA—via standardized flow cytometry of NK subpopulations, expression levels of key NKRs, or GzmB concentrations—may serve as an initial predictor of poor outcomes. Moreover, adoptive transfer of functionally mature NK cells during sustained NKA suppression could offer a therapeutic alternative for refractory septic cases [9, 13, 15, 27]. Clinical studies are already exploring NK cell-based immunotherapies for infectious disease [9, 15, 27].

To substantiate these clinical implications, we investigated how the dynamics of NK cell function differ mechanistically between distinct sepsis models. To further delineate the immunological mechanisms underlying these prognostic differences, we compared the trajectory of NK cell function and receptor expression between MGS and HGS. In MGS, NK cell-mediated immunity showed partial restoration over time, with recovery of NKA, increased expression of activating NKRs, and reduced inhibitory signals. Conversely, HGS was characterized by profound and sustained NK cell depletion, persistent NKA suppression, and dominant expression of inhibitory NKRs, consistent with immune paralysis and poor prognosis. These contrasting patterns imply that the failure to restore NK cell function in HGS may represent a key immunological determinant of adverse outcomes in refractory sepsis.

Although CD11b and CD27 are conventionally used to define NK cell maturation in mice [45], they also reflect functional specialization: CD11b⁻CD27⁺ NK cells are associated with cytokine production, whereas CD11b⁺CD27⁻ NK cells exhibit heightened cytolytic activity via GzmB and perforin [58–61]. In our study, GzmB expression patterns across these subsets supported a functional link between maturation stage and effector capacity. MGS was marked by an expansion of terminally differentiated NK cells and progressive GzmB upregulation, while HGS exhibited a predominance of immature-like NK subsets and persistently low GzmB levels. This skewing toward cytokine-producing phenotypes in HGS may contribute to sustained inflammation and immunopathology in refractory sepsis. While our analysis was phenotypic rather than functional, these subset shifts may underlie key differences in NK cell-mediated immune regulation according to sepsis severity.

Although Ly49H is best known for recognizing the MCMV glycoprotein m157 and mediating antiviral NK responses [36, 62–65], accumulating evidence indicates that its expression and signaling can also be modulated during bacterial sepsis and systemic inflammation, independent of viral infection [23, 39, 66, 67]. In our polymicrobial sepsis model, Ly49H⁺ NK cells were numerically reduced and functionally blunted in HGS, consistent with DAP12 pathway dysregulation and diminished receptor-triggered effector responses; by contrast, MGS showed progressive recovery of the Ly49H⁺ subset, indicating preserved activation under milder disease [39, 40]. We therefore used Ly49H mechanistically—as a readout of activating-circuit integrity—to test whether severe bacterial sepsis perturbs pathways that calibrate NK effector readiness [23, 39, 40]. Accordingly, Ly49H results should be interpreted within a balanced activating–inhibitory panel, rather than as a virus-specific surrogate; in parallel, Ly49D (activating) and Ly49C/G2 (inhibitory) anchor the activation–inhibition axis, contextualizing the divergent MGS recovery versus HGS persistence phenotypes [23, 39, 40, 50]. Finally, given species-specific NK receptor systems (murine Ly49 versus human KIR and selected NKG2 family members), our conclusions are framed at the pathway level—i.e., ITAM/ITIM balance, education/licensing, and repertoire remodeling during systemic inflammation—and we caution against one-to-one extrapolation of receptor-specific findings to humans [41, 43, 44, 50, 68].

Accumulating evidence indicates that NK cells exert phase-dependent, dual effects during sepsis [69]. Early after insult, activated NK cells can amplify systemic inflammation and tissue injury via rapid IFN-γ release and cytotoxic programs; in abdominal sepsis models, genetic or pharmacologic attenuation of granzyme A reduces inflammatory damage and improves survival without loss of bacterial control, underscoring a detrimental facet of early NKA [57, 70, 71]. Later, the NK compartment frequently becomes numerically and functionally compromised—with reduced circulating counts, impaired IFN-γ production, and increased expression of inhibitory checkpoints (e.g., PD-1/PD-L1, NKG2A); higher proportions of PD-L1⁺ NK cells correlate with greater illness severity and worse short-term outcomes, highlighting a clinically relevant immunosuppressive phenotype [27, 72, 73]. Within this framework, our biphasic CLP model shows that MGS exhibits an initially suppressed but recoverable NK state by day 7 (increased activating NKRs, expansion of CD11b⁺CD27⁻ subsets, higher NK counts, restoration of GzmB), whereas HGS is characterized by persistent suppression of NK activity across matched time points. These divergent trajectories suggest that recovery vs fixation of NK dysfunction may help stratify sepsis courses and align with survival differences [39]. Therapeutically, the duality of NK biology argues for phase-specific interventions—tempering excessive early NK-driven inflammation, while restoring later NK competence (e.g., judicious IL-15—based support or checkpoint modulation such as PD-1/PD-L1 or NKG2A blockade)—with careful attention to timing, endotype, and pathogen/context, and to translational limits between murine CLP and heterogeneous human sepsis [73–77].

Our CLP-based findings align with human data framing sepsis as a course from early hyperinflammation to sustained immune dysfunction, during which innate/lymphoid compartments—including NK cells—undergo quantitative loss and functional reprogramming [69]. Within this framework, the MGS trajectory in our model echoes immune reconstitution described in survivors, whereas HGS resembles the persistent immunosuppression reported in non-survivors [78]. Nonetheless, translation warrants caution: CLP standardizes a young, specific pathogen-free, intra-abdominal polymicrobial nidus, whereas clinical sepsis spans heterogeneous pathogens, comorbidities, prior antimicrobials/colonization, and variability in source control and therapy—factors that can reshape immune trajectories beyond what CLP captures [79]. Interspecies receptor biology further limits one-to-one mapping: mice rely on Ly49 receptors, while humans use KIR and CD94/NKG2 families, generating distinct education/tolerance circuits and ligand landscapes that recalibrate cytotoxic thresholds differently in sepsis [50]. These receptor-level disparities help explain the apparent paradox that human NK cell deficiencies are associated with severe infection and higher mortality, whereas selected murine settings report survival benefits from NK/effector attenuation (e.g., granzyme A deficiency or inhibition) during abdominal sepsis—differences likely reflecting immune system complexity, lifelong microbial exposures, immunosenescence, and phase-dependent NK effects [80]. Clinically, human studies document reduced NK counts/cytotoxicity in sepsis and up-regulated inhibitory checkpoints (e.g., PD-1/PD-L1, NKG2A), with higher PD-L1⁺ NK proportions correlating with greater illness severity and poorer short-term outcomes—underscoring a narrow therapeutic window that favors calibrated, context-specific modulation rather than uniform augmentation [69, 72]. Guided by this, we advocate phase-aware, patient-tailored immune monitoring that integrates phenotype (KIR/NKG2 profile, maturation state) with function (intracellular GzmB/perforin, CD107a degranulation, secretion assays, standardized killing readouts) to position individuals along an NK-immune trajectory and guide stage-appropriate interventions; prospective human cohorts should test whether CLP-derived signatures—subset skewing, receptor remodeling, effector output—define actionable endotypes and predict response to targeted immunoadjuvants [27, 79].

In this study, sepsis severity was operationalized a priori using standardized CLP settings (mid-grade vs high-grade) and evaluated by 7-day survival profiles, adopting survival as a pragmatic surrogate for host response. Our study focused on linking surgical exposure to survival, rather than re-measuring biomarker panels, because prior work has robustly mapped CLP surgical intensity to graded physiologic and biochemical responses (e.g., rises in plasma IL-6 and blood lactate, hypothermia, higher Murine Sepsis Scores) and to organ dysfunction and mortality [30, 81–84]. In keeping with animal-welfare principles (3Rs) [31]—we avoided longitudinal handling and repeated blood sampling that can perturb physiology and bias survival, and concentrated analytic power on survival-based stratification and endpoint tissue assays. This survival-anchored operationalization remains fully aligned with the Sepsis-3 framework of a dysregulated host response leading to organ dysfunction, while minimizing redundant animal procedures [1, 84]. Euthanasia was performed at predefined, IACUC-approved humane endpoints, and these murine welfare criteria are not intended to mirror routine clinical sepsis presentations. Nonetheless, in the preterminal phase of human sepsis—typically with advanced multiorgan failure—overlapping features (e.g., severe hypothermia, decreased level of consciousness, respiratory compromise) may occur, providing limited but relevant clinical context.

This study has several limitations. First, because our findings derive from a murine CLP-induced polymicrobial sepsis model, generalizability to human sepsis driven by specific pathogens or infection sites may be limited despite the model’s strong clinical relevance [30]. Second, although polyinosinic:polycytidylic acid [poly(I:C)] is a widely used positive control for NK cell activation via TLR3-mediated induction of type I interferons, IL-12, and IL-15, we did not include it in our design [85]. Third, we did not include longitudinal sham cohorts; this choice reflects our prespecified design and 3R (particularly Refinement) considerations. Fourth, the absence of comprehensive profiling across all NKRs, cytokines, and ligand interactions constrains the interpretation of the full NKA landscape in sepsis. Fifth, transcript-level measurements of inflammatory mediators (e.g., IL-6, IFN-γ) were not prospectively collected across all groups and time points, precluding matched transcript-level correlations with NK-associated markers; exploratory subset analyses would be underpowered and potentially biased. Accordingly, it would be important for future studies to incorporate prespecified, longitudinal cytokine profiling (transcript and protein) with correlation analyses aligned to NK-associated markers. Sixth, NK cell function was inferred from markers such as CD11b/CD27, Ly49 repertoires, and GzmB, which may not capture the full functional plasticity or heterogeneity. Seventh, analyses were limited to animals surviving at each time point, introducing potential survival bias, particularly at later stages (e.g., day 7 in MGS or day 3 in HGS). Eighth, bulk RT-PCR of sorted NK cells did not account for subset heterogeneity; single-cell transcriptomics could resolve subset-specific dynamics in future work. Finally, because GzmB is largely intracellular at baseline, supernatant ELISA does not quantify the cell-associated reservoir and may therefore underestimate total content. We therefore focused on a standardized secretion-focused functional assessment to interrogate released (effector) GzmB as one complementary facet of NK cell function in sepsis, while recognizing that intracellular flow cytometry (ICS) can quantify the intracellular pool at single-cell resolution, link it to maturation/NKR phenotypes (e.g., CD27/CD11b, Ly49), and map subset-level heterogeneity. Future studies will integrate ICS for GzmB, CD107a degranulation, enzyme-linked immunospot, and target-cell co-culture cytotoxicity assays (e.g., K562 or YAC-1) to triangulate reservoir–degranulation–release–killing and provide cellular-resolution corroboration of our secretion-based findings [86]. Despite these limitations, the use of sham-operated mice as negative controls, along with a well-validated CLP model and survival curves consistent with previous reports [30], supports the reliability of our experimental design and interpretations.

Given this robust framework, we further ensured analytical rigor and reproducibility through stratified modeling, comprehensive tissue analysis, and sufficient statistical power. We distinguished between MGS and HGS to model sepsis severity and comprehensively profiled NK cell subsets and receptor expression across WB and lymphoid organs. A relatively large initial sample size ensured sufficient statistical power, particularly at later time points, enabling the detection of subtle immunological differences. Unlike clinical studies, where precise sepsis onset and serial immune sampling are difficult, our animal model allowed dynamic tracking of NKA from defined time points after sepsis induction. Moreover, the dynamic, severity-dependent expression of Ly49 family receptors in NK cells remains underexplored in sepsis research. This scarcity of prior evidence reinforces the novelty and relevance of our findings and provides a solid foundation for future investigations into NK cell-mediated immune responses in sepsis.

Taken together, our findings indicate that persistent NKA suppression in critical sepsis is driven by multiple converging factors—including reduced NK cell numbers, inhibitory receptor dominance, impaired cytolytic capacity, and skewed subset composition—culminating in immune dysregulation. These alterations were consistently observed in both WB and lymphoid tissues, supporting the robustness of our conclusions. Given the limited options for targeted sepsis therapies, modulating NK cell function—either via receptor reprogramming or adoptive NK cell transfer—may represent a promising strategy, particularly in patients with high APACHE II scores or early-organ dysfunction. Further mechanistic studies are warranted to elucidate the molecular regulators underlying these NK cell alterations.

## Conclusion

In fatal sepsis, we observed persistent alterations in NKRs’ expression—namely increased inhibitory and decreased activating forms—together with marked changes in NK cell subsets. Although our analysis was phenotypic rather than functional, these patterns suggest an association between NK cell dysregulation and poor outcomes. Future work incorporating direct functional assays will be essential to confirm these findings and clarify their mechanistic and translational significance.

## Supplementary Information

Supplementary material 1.

## Data Availability

Data supporting the findings of this study are available from the corresponding author upon reasonable request.

## Abbreviations

ANOVA: Analysis of variance
AO: Acridine orange
BM: Bone marrow
CD: Cluster of differentiation
CLP: Cecal ligation and puncture
Ct: Cycle threshold value
DAP: DNAX-activating protein
ELISA: Enzyme-linked immunosorbent assay
FACS: Fluorescence-activated cell sorting
GAPDH: Glyceraldehyde 3-phosphate dehydrogenase
GzmB: Granzyme B
HGS: High-grade sepsis
IACUC: Institutional Animal Care and Use Committee
Ig: Immunoglobulin
IFN-γ: Interferon-γ
IL: Interleukin
IQR: Interquartile range
ITAM: Immunoreceptor tyrosine-based activation motif
ITIM: Immunoreceptor tyrosine-based inhibition motif
LNs: Lymph nodes
Ly: Lymphocyte antigen
MCMV: Murine cytomegalovirus
MGS: Mid-grade sepsis
mRNA: Messenger RNA
NK: Natural killer cell
NKA: Natural killer cell activity
NKR: Natural killer cell receptor
PI: Propidium iodide
Q: Quartile
RT-PCR: Real-time polymerase chain reaction
WB: Whole blood
