# Supplementary material for: Dynamic changes of natural killer cell immunophenotypes and receptors according to the mortality in the intra-abdominal murine sepsis model
Source: Intensive Care Med Exp. 2025 Nov 19;13:117. doi: 10.1186/s40635-025-00829-6 (PMC12630530; doi:10.1186/s40635-025-00829-6)
Supplement: Supplementary file 1 — Supplementary material 1. [file 40635_2025_829_MOESM1_ESM.docx]

Supplementary Material

**Manuscript Title:** *Dynamic Changes of Natural Killer Cell Immunophenotypes and Receptors According to the Mortality in the Intra-Abdominal Murine Sepsis Model*

**Table S1.** Overview of cell populations used for quantitative flow cytometric and molecular analyses per experiment and figure.

| **Figure** | **Method** | **Cell populations** | **Total number of cells or events** |
| --- | --- | --- | --- |
| **Fig. 1** Overview of the experimental workflow using the CLP-induced sepsis mouse model, including subgroup allocation and the gating strategy applied for flow cytometric analysis | Flow cytometry | **All events recorded**  (Not sorted) | 1,000,000 |
| **Fig. 2** Dynamic alterations in NK cell populations over time in relation to sepsis severity | Flow cytometry | **Total lymphocytes**  (Not sorted) | 100,000^a^ |
| **Table 1.** Comparison of dynamic changes in CD11b⁺ and CD27⁺ NK cell subpopulations between mid-grade and high-grade sepsis | Flow cytometry | **Gated CD3^-^/NK1.1^+^ lymphocytes**  (Total NK cells)  (Not sorted) | 5,000^a^ |
| **Fig. 3** Differential mRNA expression of inhibitory and activating NK cell receptors in in a murine model of mid-grade and high-grade sepsis | RT-PCR | **Sorted NK cells**^b^ | 1,000  (A total of 20 ng of RNA) |
| **Fig. 4** Boolean analysis of NK cell receptor expression across sepsis groups | Flow cytometry | **Gated CD3^-^/NK1.1^+^ lymphocytes**  (Total NK cells)  (Not sorted) | 5,000^a^ |
| **Fig. 5** Plasma concentrations of granzyme B and interferon-γ, and relative mRNA expression of granzyme B in various organs | | | |
| (**A**) Plasma concentrations of granzyme B and interferon-γ | ELISA | **Sorted NK cells**^b^ | 1,000 |
| (**B**) Relative mRNA expression of granzyme B | RT-PCR | **Sorted NK cells**^b^ | 1,000  (A total of 20 ng of RNA) |
| **Fig. S1** Dynamic alterations in the total NK cell population among lymphocytes following mid-grade and high-grade sepsis, in comparison with the sham group. | Flow cytometry | **Total lymphocytes**  (Not sorted) | **—** |
| **Fig. S2** Comparisons of NK1.1 mRNA expression according to sepsis lethality and time elapsed after sepsis induction in whole blood, bone marrow cells, liver, spleen, and lymph nodes | RT-PCR | **Sorted NK cells**^b^ | 1,000  (A total of 20 ng of RNA) |
| **Fig. S3** Flow cytometric comparison of dynamic changes in CD11b⁺ and CD27⁺ NK cell subpopulations between mid-grade and high-grade sepsis | Flow cytometry | **Gated CD3^+^/NK1.1^+^ lymphocytes**  (**Total NK cells**)  (Not sorted) | 5,000^a^ |
| **Fig. S4** Flow cytometric comparison of dynamic changes in NK cell subsets expressing Ly49D/H (activating) and Ly49C/G2 (inhibitory) receptors between mid-grade and high-grade sepsis | Flow cytometry | **Gated CD3^+^/NK1.1^+^ lymphocytes**  (**Total NK cells**)  (Not sorted) | 5,000^a^ |

^a^Indicates the total number of cells used as the denominator for quantitative analysis. ^b^Represents purified NK cells isolated by FACS from the total acquired cell population.

Aberrations: CD, cluster differentiation; CLP, cecal ligation and puncture; ELISA, enzyme-linked immunosorbent assay; FACS, fluorescence-activated cell sorting; HGS, high-grade sepsis; LN, lymph nodes; MGS, mid-grade sepsis; mRNA, messenger RNA; NK, natural killer cell; RNA, ribonucleic Acid; RT-PCR, reverse transcription polymerase chain reaction.


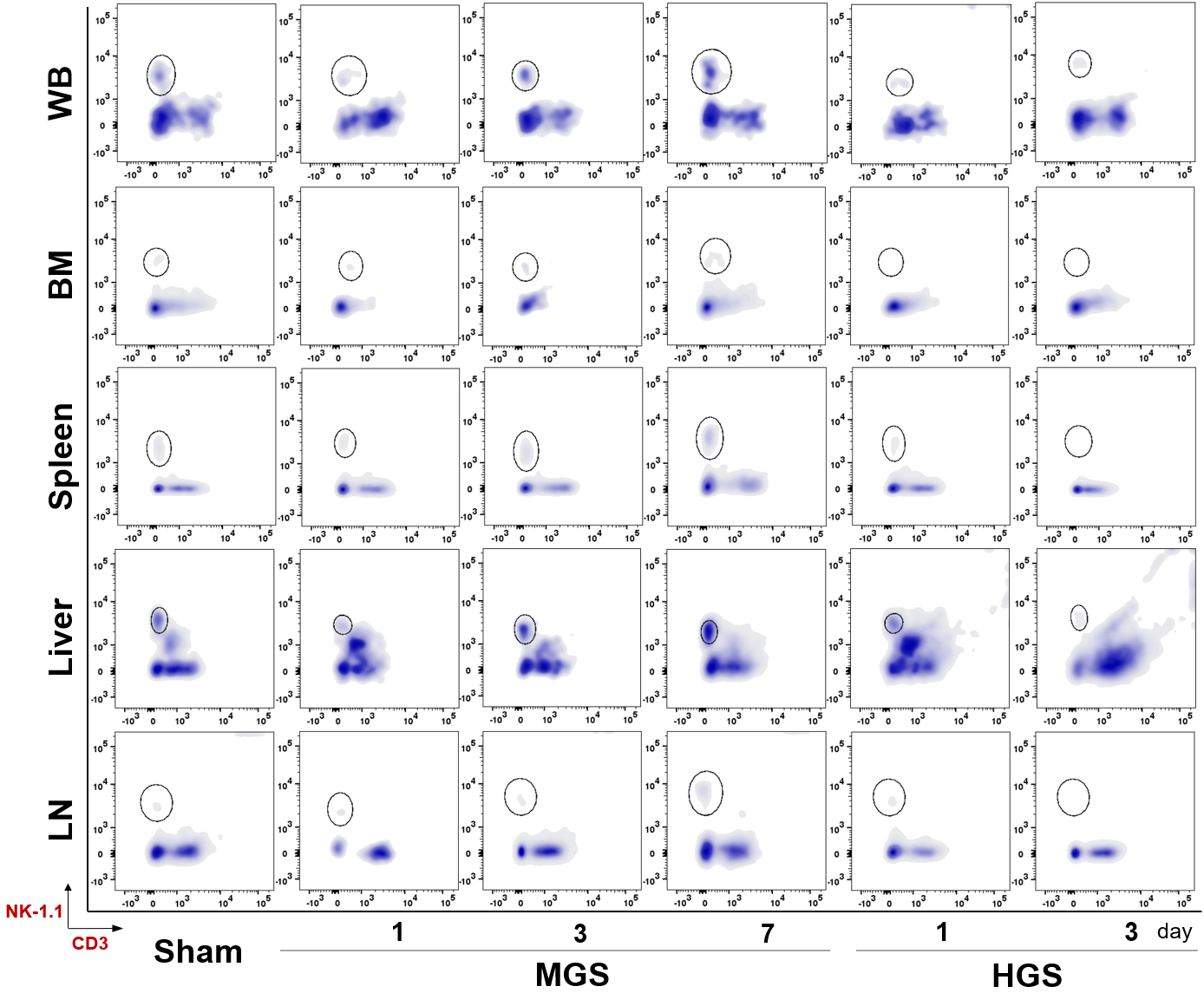
**Fig. S1.** Dynamic alterations in the total NK cell population among lymphocytes following mid-grade and high-grade sepsis, in comparison with the sham group.

Representative flow cytometry plots from each sample were selected and illustrated to highlight the most distinct differences between the groups. Distinct NK cell populations (CD3^-^/NK1.1^+^) are indicated by circles. In the HGS group, NK cells were scarcely detectable—representing less than 3% of total lymphocytes—on day 1 post-sepsis induction in the BM, and on day 3 in the BM, spleen, and LN.

Abbreviation: BM, bone marrow cell; CD, cluster differentiation; HGS, high-grade sepsis; LN, lymph node; MGS, mid-grade sepsis; NK, natural killer cell; WB, whole blood.


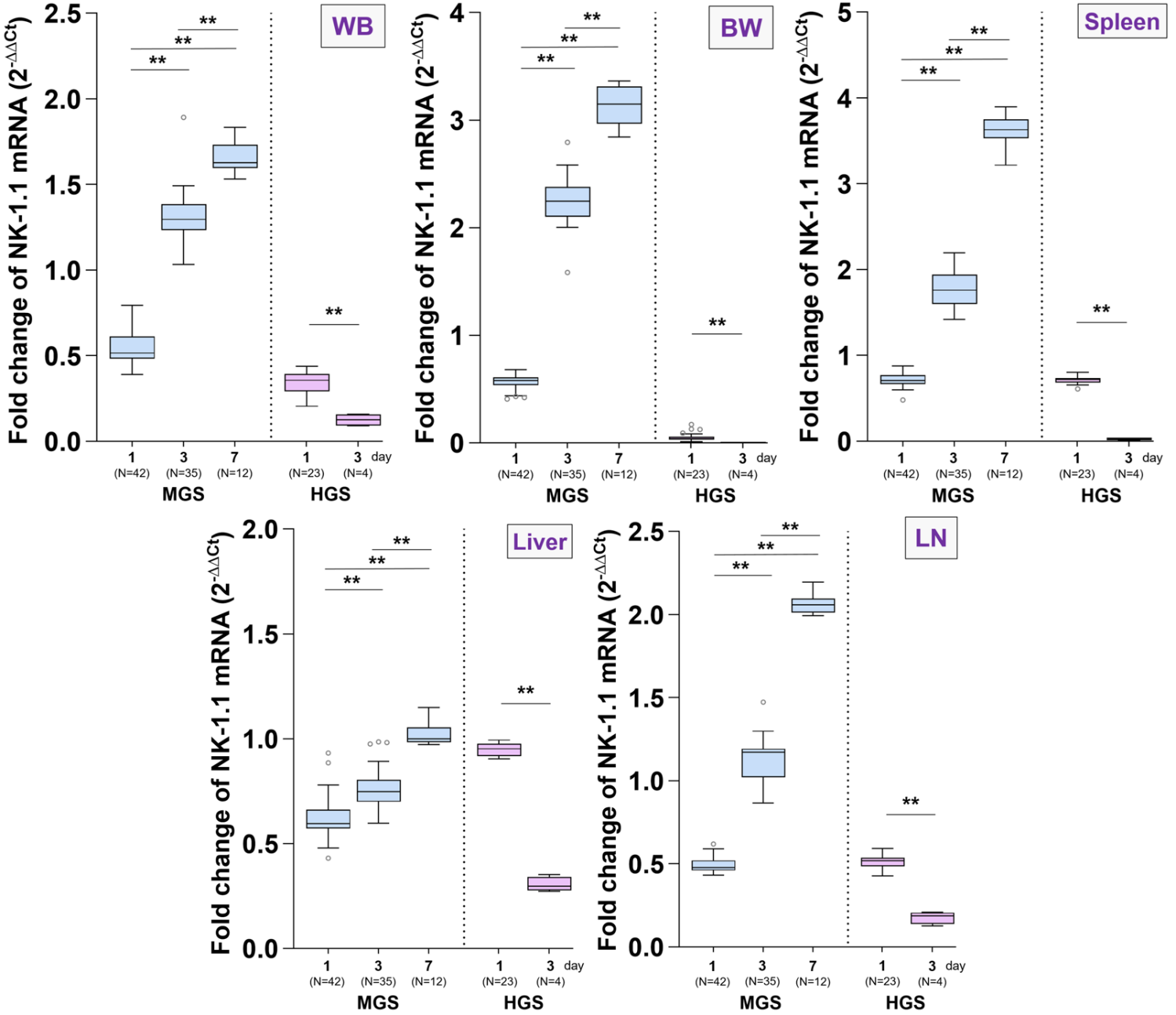
**Fig. S2.** Comparisons of NK1.1 mRNA expression according to sepsis lethality and time elapsed after sepsis induction in whole blood, bone marrow cells, liver, spleen, and lymph nodes.

These figures represent a comparison of NK1.1-encoding mRNA expression levels at different time points in mid-grade sepsis (MGS) and high-grade sepsis (HGS) groups, as measured by RT-PCR. For objective quantitative analysis and valid statistical comparison, a total of 2,000 purified NK cells were obtained by FACS sorting, and 20 ng of total RNA extracted from these cells was used for the RT-PCR reaction. Each graph is shown as a box-and-whisker plot using the Tukey method. The central line within the box represents the median (Q2), while the lower and upper edges of the box correspond to the 25th (Q1) and 75th (Q3) percentiles (interquartile range [IQR]: Q1–Q3), respectively. The whiskers extend to values within the range of Q1 − 1.5 × IQR and Q3 + 1.5 × IQR (where IQR = Q3 − Q1). Data points beyond this range are displayed as individual dots, representing statistical outliers. The number of samples per group is indicated in the figure. A total of 27 samples were obtained from the sham group. In the MGS group, 42, 35, and 12 samples were collected on days 1, 3, and 7 after CLP surgery, respectively. In the HGS group, 23 and 4 samples were collected on days 1 and 3, respectively; no samples were available on day 7, as all mice in this group had died by that time, making specimen collection and analysis impossible. Statistical comparisons were performed using two-way ANOVA with Bonferroni post hoc correction in the MGS group and two-tailed unpaired Mann–Whitney U test in the HGS group. ^*^*P* < 0.01, ^**^*P* < 0.001.

Abbreviation: ANOVA, Analysis of Variance; BM, bone marrow cell; CD, cluster differentiation; CLP, cecal ligation and puncture; Ct, Cycle threshold; FACS; fluorescence-activated cell sorting; HGS, high-grade sepsis; IQR, interquartile range; LN, lymph nodes; MGS, mid-grade sepsis; mRNA, messenger RNA; NK, natural killer cell Q, quartile; RT-PCR, reverse transcription polymerase chain reaction; WB, whole blood.


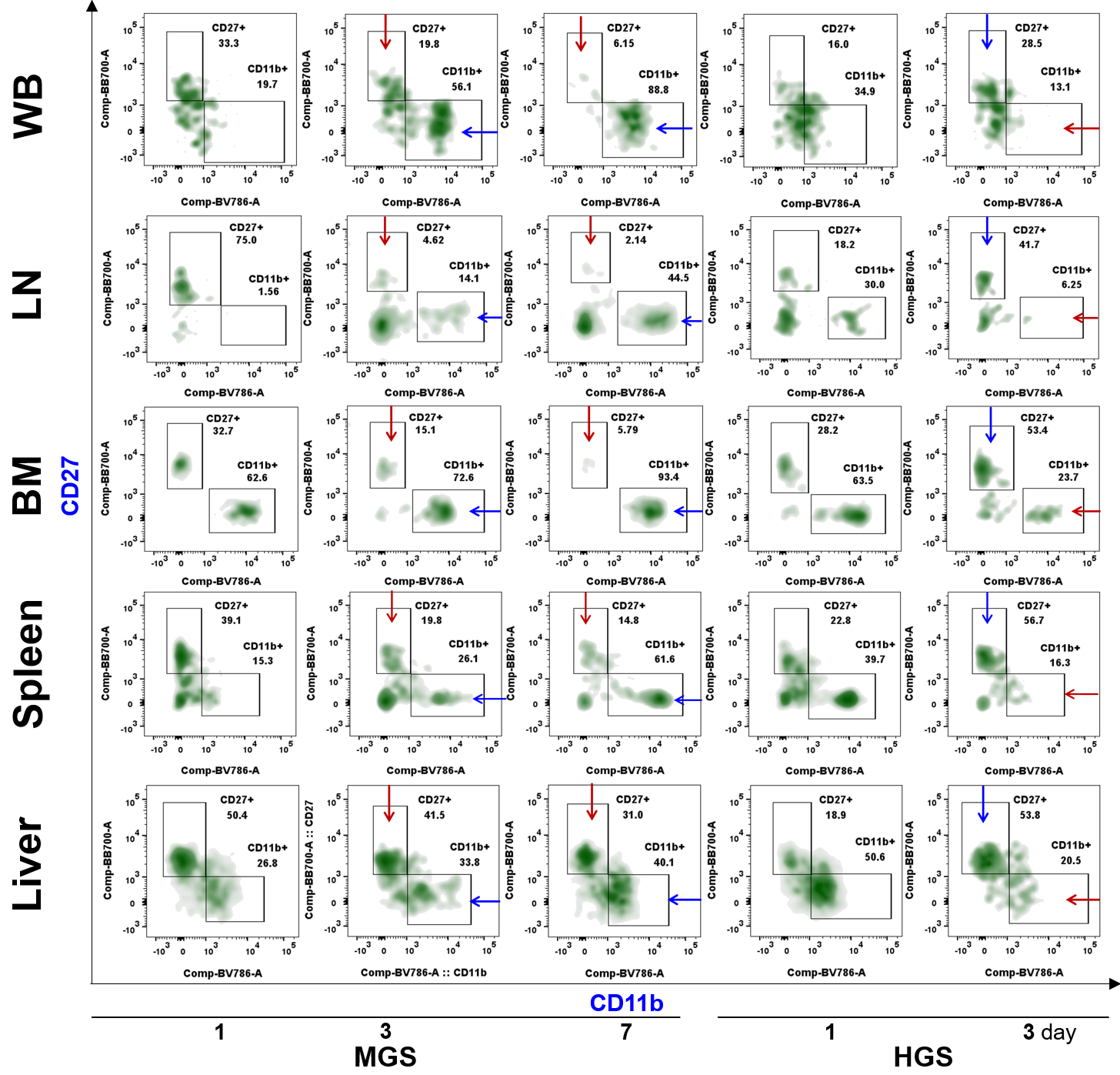
**Figure S3.** Flow cytometric comparison of dynamic changes in CD11b⁺ and CD27⁺ NK cell subpopulations between mid-grade and high-grade sepsis.

Representative flow cytometry plots from each sample were selected and illustrated to highlight the most distinct differences between the groups. To ensure consistent quantification of NK cell subpopulations expressing CD11b alone or CD27 alone in each flow cytometry plot, the percentages were calculated using a denominator of 5,000 gated CD3⁻/NK1.1⁺ lymphocytes (total NK cells). Samples from the sham group were not included in the figure, as all exhibited double-negative expression for both CD11b and CD27. In both the MGS and HGS groups, the CD11b⁺/CD27⁺ double-positive NK cell subset was consistently measured at less than 5% in most cases, indicating a relatively low frequency. The blue and red arrows in each flow cytometry plot indicate an increase or decrease, respectively, in comparison to the first sample (day 1 post-CLP surgery) within the corresponding group (MGS or HGS).

Abbreviation: BM, bone marrow cell; CD, cluster differentiation; CLP, cecal ligation and puncture; HGS, high-grade sepsis; LN, lymph nodes; MGS, mid-grade sepsis; NK, natural killer cell WB, whole blood.

**Fig. S4**. Flow cytometric comparison of dynamic changes in NK cell subsets expressing Ly49D/H (activating) and Ly49C/G2 (inhibitory) receptors between mid-grade and high-grade sepsis


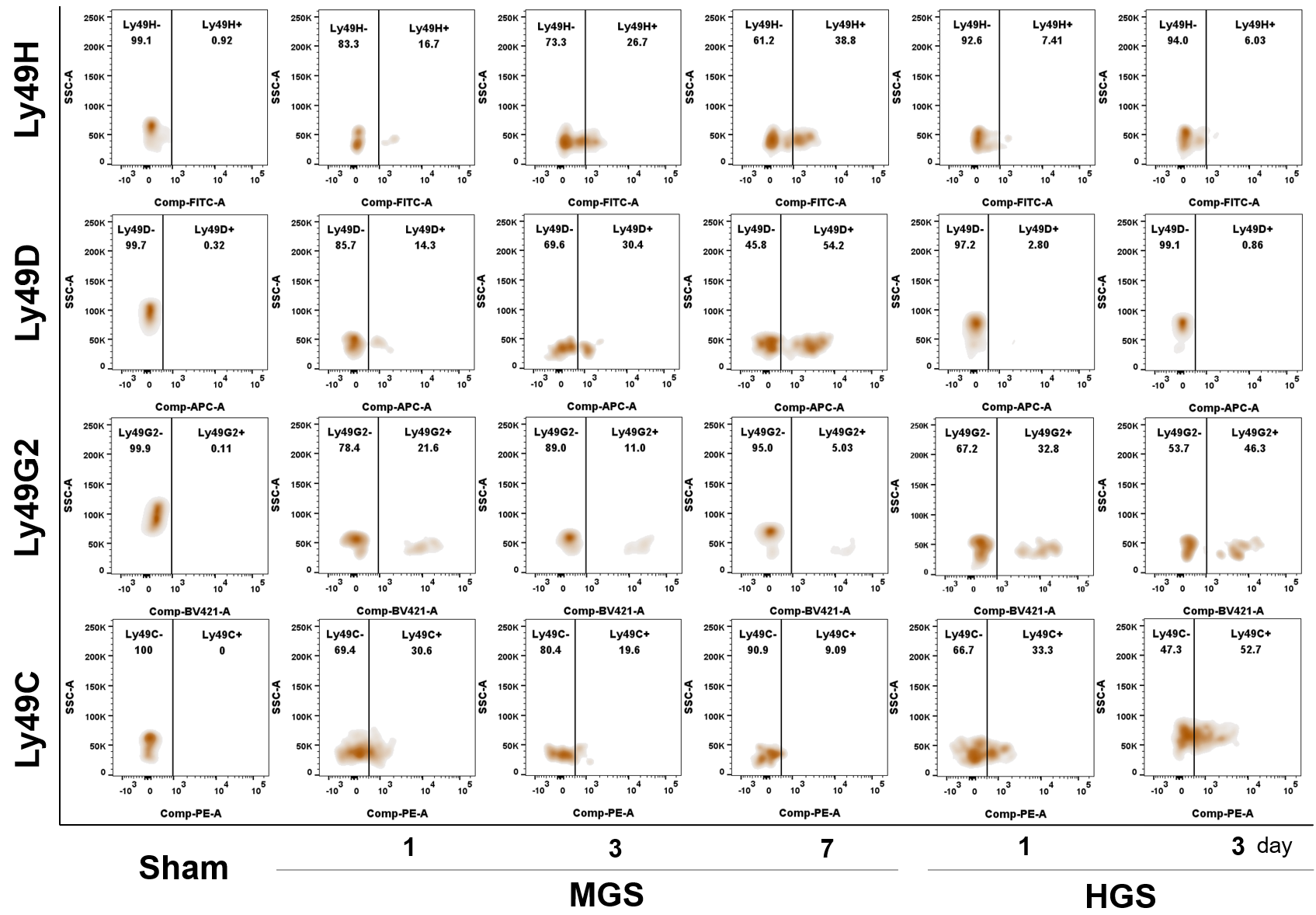


Representative flow cytometry plots from each group were selected to highlight the most distinct differences in NK cell receptor expression. Flow cytometry plots depict NK cell subsets expressing positive or negative combinations of the four receptors—Ly49H, Ly49D (activating), and Ly49G2, Ly49C (inhibitory)—as identified through Boolean gating analysis. Each dot plot indicates the percentage of NK cells expressing (right of the vertical threshold) or not expressing (left) the indicated receptor. For consistent quantification of NK cell subpopulations expressing each receptor individually, the percentage of positive cells in each plot was calculated based on 5,000 gated CD3⁻/NK1.1⁺ lymphocytes (i.e., total NK cells). In the sham group, the proportion of NK cells positive for each individual receptor was consistently below 2% in all plots.
